# Hemoadsorption in acute respiratory distress syndrome patients requiring venovenous extracorporeal membrane oxygenation: a systematic review

**DOI:** 10.1186/s12931-024-02675-8

**Published:** 2024-01-12

**Authors:** Wenli Li, Yuansen Chen, Duo Li, Xiangyan Meng, Ziquan Liu, Yanqing Liu, Haojun Fan

**Affiliations:** 1 Institute of Disaster and Emergency Medicine, Tianjin University, Tianjin, China; 2 Wenzhou Safety (Emergency) Institute, Tianjin University, Wenzhou, China

**Keywords:** Hemoadsorption, Venovenous extracorporeal membrane oxygenation, Acute respiratory distress syndrome, Inflammation, Mortality, Hemodynamic stability

## Abstract

**Background:**

Venovenous extracorporeal membrane oxygenation (VV ECMO) has been widely used for severe acute respiratory distress syndrome (ARDS) in recent years. However, the role of hemoadsorption in ARDS patients requiring VV ECMO is unclear.

**Methods:**

Therefore, we conducted a systematic review to describe the effect of hemoadsorption on outcomes of ARDS patients requiring VV ECMO and elucidate the risk factors for adverse outcomes. We conducted and reported a systematic literature review based on the principles derived from the Preferred Reporting Items for Systematic Reviews and Meta-Analyses (PRISMA) statement. The systematic review searched Embase, CINHAL, and Pubmed databases for studies on ARDS patients receiving hemoadsorption and VV ECMO. The demographic data, clinical data and biological data of the patients were collected.

**Results:**

We ultimately included a total of 8 articles including 189 patients. We characterized the population both clinically and biologically. Our review showed most studies described reductions in inflammatory markers and fluid resuscitation drug dosage in ARDS patients with Coronavirus disease 2019 (COVID-19) or sepsis after hemoadsorption.

**Conclusion:**

Because most of the studies have the characteristics of high heterogeneity, we could only draw very cautious conclusions that hemoadsorption therapy may enhance hemodynamic stability in ARDS patients with COVID-19 or sepsis receiving VV ECMO support. However, our results do not allow us to draw conclusions that hemoadsorption could reduce inflammation and mortality. Prospective randomized controlled studies with a larger sample size are needed in the future to verify the role of hemoadsorption in ARDS patients requiring VV ECMO.

**Supplementary Information:**

The online version contains supplementary material available at 10.1186/s12931-024-02675-8.

## Introduction

**Fig. 1:**
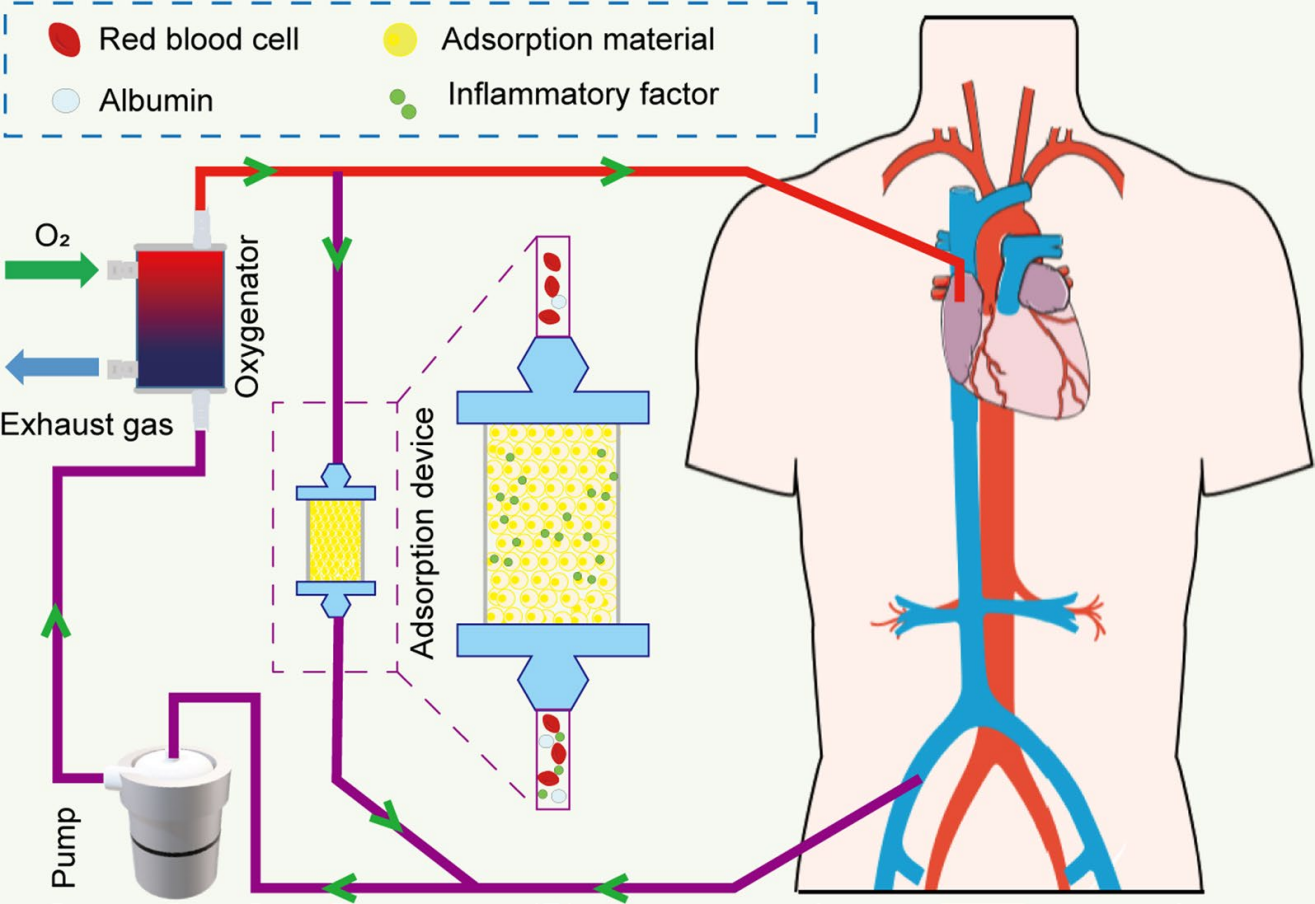
Schematic diagram of VV ECMO combined with hemoadsorption device to filter cytokines

Acute respiratory distress syndrome (ARDS) is an acute respiratory illness characterised by severe hypoxaemia and respiratory distress due to noncardiogenic pulmonary oedema [1–5]. Extracorporeal membrane oxygenation (ECMO) has been widely used due to the increase of severe ARDS patients, which can effectively improve the survival and blood oxygenation of ARDS patients compared with traditional mechanical ventilation [6–10]. Studies have shown that severe pneumonia, sepsis and other diseases that develop into ARDS are associated with uncontrolled cytokine storm [11–13]. Cytokine storm is a vicious cycle of cytokine mediated accumulation and infiltration of large numbers of immune cells, culminating in a cytokine storm that causes damage to various organ functions [14–16]. In recent years, hemoadsorption appears to offer a promising new option for the treatment of patients with an overwhelming inflammatory response leading to faster hemodynamic and metabolic stabilization [17–20]. It is an in vitro therapeutic strategy for quick and effective adsorption of cytokines, myoglobin or bilirubin in patients through porous polymer beads [21–25]. The hemoadsorption device can not only be used alone, but also can be used in combination with ECMO as a bypass circuit integrated in the ECMO circuit for blood perfusion (Fig. 1). Moreover, some adsorption materials can also remove endotoxin or other harmful substances, relieve inflammation and further disease progression [26–28]. But the role of hemoadsorption in ARDS patients requiring venovenous ECMO (VV ECMO) is unclear. Some clinical case series studies suggest that hemoadsorption is beneficial to ARDS patients requiring VV ECMO and can effectively reduce mortality and inflammatory factors [29–33]. However, some studies shown that it may not have a positive impact on ARDS patients requiring VV ECMO [34, 35]. Moreover, Coronavirus disease 2019 (COVID-19) guidelines of Extracorporeal Life Support Organization (ELSO) do not recommend the use of hemoadsorption therapy outside of clinical trials [36]. This makes it difficult for clinicians to decide whether to use hemoadsorption in ARDS patients requiring VV ECMO. Therefore, we attempted to systematically review the existing literatures to assess the effect of hemoadsorption in ARDS patients requiring VV ECMO from multiple databases. We mainly summarized clinical and biochemical outcome data and identified risk factors for adverse outcomes to guide future clinical decision and large-sample prospective studies.

## Methods

### Search strategy and selection criteria

We followed the methodology recommended by the PRISMA statement for reporting systematic reviews and the PRISMA checklist is presented in Additional file 1 and 2 [37]. We searched the Embase, CINHAL, Pubmed databases to identify relevant articles from inception to July 29, 2022. Our search was constructed by three sets of terms. The first group includes:“ards” OR “acute respiratory distress syndrome” OR “covid‐19”; the second group: “ecmo” OR “extracorporeal membrane oxygenation” OR “ecls” OR “extracorporeal life support”; the third group: “blood purification” OR “cytosorb” OR “cytokine adsor*” OR “toraymyxin” OR “endotoxin” OR “polymyxin” OR “hemoperfusion”. We also reviewed all references included in the study and comment articles related to this topic. Two authors independently identified studies that met the inclusion criteria based on the title and abstract. The included studies (including the meeting summary) were then further reviewed. Retrospective studies, prospective studies, case reports and randomized controlled trials (RCTs) were included. We excluded animal studies and overlapping studies. The inclusion criteria were pre-specified according to the PICOS method (Table 1). We also excluded reviews, editorials, and letters to editors without controlled case studies. A case series of more than 5 patients was included in the study. Language restrictions applied: We only read manuscripts of articles published in English.

**Table 1:** PICOS criteria

PICOS criteria	Description
Population	ARDS patients requiring VV ECMO
Intervention	Hemoadsorption
Comparison	Treated with VV ECMO but not receiving hemoadsorption
Outcome	Clinical and biological outcomes
Study design	Prospective and retrospective studies; randomized controlled trial; case series reporting (≥ 5 Patients)

*PICOS* Population, intervention, comparison, outcome, and Study design; *ARDS* acute respiratory distress syndrome; *VV ECMO* venovenous extracorporeal membrane oxygenation

### Data collection and risk of bias assessment

We used pre-designed tables to collect data and contact the authors for data if necessary. Data was extracted as follows: article characteristics (authors, year of publication, type of study design, country of origin), demographic data (including number of patients, age, gender, etc.), clinical outcomes and biological variables. We use the appropriate Joanna Briggs Institute checklist to assess a study risk of bias in Additional file 3 [38]. Data collection and risk assessment were conducted independently by two reviewers (WL and YC). Resolution of the inconsistency problem involves one senior author (HF).

### Analysis of clinical outcomes

The clinical indicators we focused on mainly included severity of lung injury (simplified acute physiology II score (SAPS-Score), Sequential Organ Failure Assessment (SOFA) score, the Respiratory Extracorporeal Membrane Oxygenation Survival Prediction score (RESP score)), respiratory parameters (oxygenation index), and duration of ECMO support. In addition, there were length of intensive care unit (ICU) stay and mortality. We mainly summarized the results of ARDS patients in two groups (VV ECMO, VV ECMO and hemoadsorption) through descriptive statistics.

### Biological variables

Regarding biological indicators, we mainly described the changes of inflammatory markers in ARDS patients requiring VV ECMO with/without hemoadsorption. It mainly includes the level of interleukin 6 (IL-6), C-reactive protein (CRP), procalcitonin (PCT) and lactic acid levels in the patients' blood. We also described hemodynamic parameters by the drugs used (the dose of norepinephrine). We summarize these results mainly through descriptive statistics.

## Results

### Search results

**Fig. 2:**
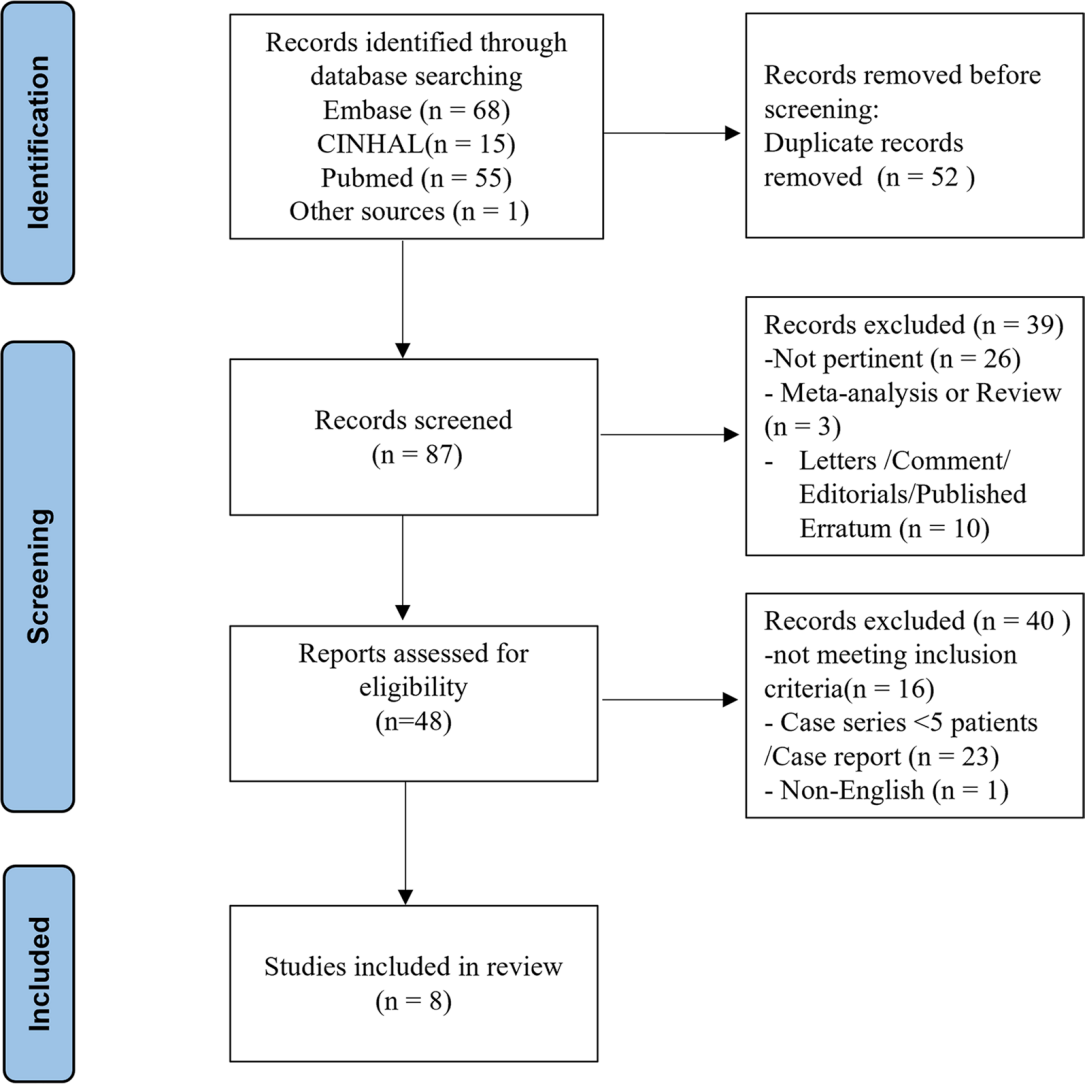
PRISMA flowchart of study identification for Systematic Reviews

Our systematic review shows that a total of 139 articles matched the inclusion criteria. As shown in the flow chart of Fig. 2, eighty-seven articles were included after removing duplicates. After the full-texts of these articles was evaluated, eight articles met the PICOS criteria and were finally selected [32, 33, 35, 39–43]. In the eight included articles, a specific comparison of patient outcomes between receiving VV ECMO and receiving VV ECMO with hemoadsorption as two organ support modalities was the main objective.

### Description of included studies

The included studies were published between 2020 and 2022 and evaluated a total of 189 participants (range 7–52 per study), with 68.3% of male patients. And 29% of patients (n = 54) with a median age of 55.3 years were supported with VV ECMO and 71% with a median age of 51.9 years were supported with VV ECMO and hemoadsorption (n = 135). The etiology of the patients in most studies was COVID-19 or sepsis, the in vitro cytokine adsorber used by patients in these studies was CytoSorb© (CytoSorbents, Princeton, NJ, USA). The reason for this phenomenon is unclear, which may be related to the emergency use authorization (EUA) issued by the Food and Drug Administration (FDA) for this product during the COVID-19 pandemic. The duration of hemoadsorption is generally 72 h, with columns replaced every 24 h. Moreover, these studies were carried out in European countries and North America (Germany, France and the United States), including 6 original research articles, one case series and a correspondence containing patients’ treatment data. The types of study design are described as followed in Table 1: four retrospective researches, one prospective randomized study, one case series, one retrospective registry and one randomized controlled trial. No meta-analysis was performed due to the small number of studies and incomplete data. Their clinical and biological characteristics were described in Table 2 and Table 3.

**Table 2:** Clinical data of the included studies in the systematic review

Author	Study design	Country	No	Cause	Age, years (Control vs Cytosorb)	Male, No (Control vs Cytosorb)	severity of disease of baseline (Control vs Cytosorb)	Pao2/Fio2 of baseline (Control vs Cytosorb)	duration of ECMO support, days (Control vs Cytosorb)	ICU length, days (Control vs Cytosorb)	Mortality, % (Control vs Cytosorb)
Akil et al.[40]	Retrospective study	Germany	Control:10 Cytosorb:16	COVID–19	62.2 ± 8.4 vs 58.5 ± 11.7	8 vs 10	SAPS II score: 44.2 ± 10.7 vs 59.3 ± 9.1	85.8 ± 15.3 vs 103.4 ± 36.5	14.8 ± 12.0 vs 17.3 ± 17.1	15.5 ± 11.6 vs 26.5 ± 20.7	30% vs 37.5% (90–day mortality)
Akil et al.[39]	Retrospective study	Germany	Control:7 Cytosorb:13	sepsis	61 ± 2 vs 61 ± 3	2 vs 5	SAPS II score: 49.5 ± 2.2 vs 58.3 ± 2.13	–	19 ± 3 vs 8 ± 2	26 ± 5 vs 26 ± 6	57% vs 0% (30–day mortality)
Rieder et al. [41]	Retrospective study	Germany	Control:9 Cytosorb:9	unrestricted	43.7 ± 13.4 vs 43.2 ± 13	6 vs 7	SOFA score: 15 ± 7 vs 17 ± 2 RESP score: 1 ± 4 vs –7 ± –4.5 PRESERVE score: 4 ± 3.5 vs 6 ± 1.5	–	–	20.5 ± 7 vs 36 ± 75.5	77.8% vs 44.4% (ICU mortality)
Supady et al. [35]	Randomized controlled trial	Germany	Control:17 Cytosorb:17	COVID–19	59.0 (43.5–66.5) vs 62.0 (54.0–71.5)	13 vs 12	SOFA score: 9.0 (7.0–10.5) vs 9.0 (8.0–10.0) RESP score: 1.0 (0–3.5) vs 1.0 (0.5–2.0) PRESERVE score: 4.0 (2.0–6.0) vs 4.0 (3.0–5.0)	84.2 (59.9–95.6) vs 62.7 (48.5–72.7)	–	–	24% vs 82% (30–day mortality)
Lebreton et al. [42]	Prospective study	France	Control:11 Cytosorb:11	COVID–19	49(33–65)	16	SAPSII score: 46 (17–92)	–	20(1–42) vs 25(6–56)	–	36.4% vs 27.3% (60–day mortality)
Kogelmann et al. [32]	Case series	Germany	7	sepsis	51. ± 8.6	7	SAPS score: 47.6 ± 8.6 SOFA score: 13.4 ± 1.7	89(range:55.5–120)	8.7 ± 4	25.6 ± 17.8	42.9% (ICU mortality)
Song et al. [43]	Retrospective Registry	USA	52	COVID–19	48(43–55)	34	SOFA score: 6.9 ± 3.87	–	31.6 ± 25.5	–	30.8% (ICU mortality) 17.3% (30–day mortality) 26.9% (90–day mortality)
Geraci et al. [33]	Retrospective study	USA	10	COVID–19	45(37–51)	9	–	85 (65–106)	22 (13–64)	47 (34–88)	10% (90–day mortality)

*USA* The United States of America; *No* the number of patients; *Control* VV ECMO; *Cytosorb* VV ECMO + hemoadsorption; SAPS II score: the simplified acute physiology score II score; *SOFA score* the Sequential Organ Failure Assessment score; *RESP score* the Respiratory Extracorporeal Membrane Oxygenation Survival Prediction score

**Table 3:** Biochemical data of the included studies in the systematic review

Author	IL–6, pg/ml (Control vs Cytosorb)	CRP, mg/dl (Control vs Cytosorb)	Procalcitonin, ng/ml (Control vs Cytosorb)	Lactate, mmol/L (Control VS Cytosorb)	Drug, μg/Kg/min (Control vs Cytosorb)
Akil et al. [40]	Baseline:134.8 ± 101.9 vs1067.9 ± 1276.9	–	Baseline: 1.4 ± 2.7 vs 16.8 ± 60.9	Baseline: 1.9 ± 0.7 vs 2.4 ± 1.1 72 h: 1.2 vs 1.7	Norepinephrine: Baseline: 0.05 ± 0.04 0.2 ± 0.1
Akil et al. [39]	–	Baseline: 27.2 ± 2.9 vs 35 ± 5 12 h: 29 ± 3.3 vs 24 ± 3 24 h: 25.01 ± 2.8 vs 16 ± 3 48 h: 22.6 ± 3.1vs 12 ± 3 72 h: 20.02 ± 2.4 vs 10 ± 3	Baseline: 13.24 ± 9.7 vs 15.6 ± 5.4 12 h: 18.95 ± 16.32 vs 9.05 ± 3.9 24 h: 12.5 ± 10.5 vs 4.71 ± 2.3 48 h: 8.14 ± 5.9 vs 2.71 ± 1.5 72 h: 4.1 ± 3.4 vs 2.3 ± 1.2	Baseline: 2.7 ± 0.34 vs 4.1 ± 0.97 12 h: 1.7 ± 0.55 vs 2.1 ± 0.57 24 h: 1.57 ± 0.5 vs 1.6 ± 0.48 48 h: 1.7 ± 0.29 vs 1.3 ± 0.37 72 h: 2 ± 0.37 vs 1.1 ± 0.3	Norepinephrine: Baseline: 0.603 ± 0.08 vs 0.83 ± 0.16 12 h: 0.6 ± 0.13 vs 0.19 ± 0.04 24 h: 0.47 ± 0.14 vs 0.045 ± 0.01 48 h:0.38 ± 0.11 vs 0.009 ± 0.005
Rieder et al. [41]	–	–	–	–	–
Supady et al. [35]	Baseline: 289.0(84.7–787.0) vs 357.0(177.4–1186.0) 72 h:112.0 (48.7–198.5) vs 98.6 (71–192.8)	Baseline:16.93 (12.86–34.22) vs 25.49 (14.8–37.44)	Baseline: 1.34 (0.37–5.98) vs 0.73 (0.50–1.84)	Baseline: 1.4 (0.9–1.8) vs 1.8(1.2–2.3) 72 h:1.25(0.93–1.85) vs 1.35(1.05–1.58)	Norepinephrine: Baseline: 0.03(0.00–0.36) vs 0.15(0.04–0.22) 72 h: 0(0–0.1) vs 0.07(0.03–0.13)
Song et al. [43]		Baseline: 14.4 ± 18.91 72 h: 9.8 ± 9.0–		–	–
Geraci et al. [33 ]	Baseline: 22 (9–618) 72 h:11 (7–146)	Baseline: 117 (31–263) 72 h: 64 (7.8–105)	Baseline: 1.2 (0.21–3.8) 72 h: 0.19 (0.08–15)	Baseline: 1.60 (1.32–2.55) 72 h:1.35 (1.08–1.53)	–

*Control* VV ECMO; *Cytosorb* VV ECMO + hemoadsorption; *CRP* C-reactive protein; *IL-6* interleukin-6

### A comparison of clinical outcomes between two groups

Mortality for ARDS patients (mainly COVID ARDS patients) supported with VV ECMO (control group) ranged from 30% to 77.8%. Mortality for ARDS patients supported with a combination of VV ECMO and hemoadsorption (cytosorb group) ranged from 0 to 82%. Three of the five studies on comparing survival rates between the two groups concluded that hemoadsorption was associated with high survival rates [32, 39, 41], one study found that hemoadsorption was associated with low survival^35^]. Six studies showed the average VV ECMO duration, of which only 3 studies compared the data of the control group and the cytosorb group, while the other 3 studies only showed the data of the cytosorb group. Two studies showed that the mean duration of VV ECMO in the cytosorb group and the control group was 17.3 ± 17.1 days vs 14.8 ± 12.0 days and 25(6–56) days vs 20 (1–42) days, respectively [40, 42]. However, another study found that the mean VV ECMO duration in the cytosorb group was higher than that in the control group: 8 ± 2 days vs 19 ± 3 days [39]. Unfortunately, our results do not allow us to draw conclusions about whether treatment with hemoadsorption can reduce the duration of ECMO. In addition, two of the studies showed that cytosorb group had a longer ICU time. Akil and colleagues’ study found mean ICU length of stay was 15.5 ± 11.6 days in the control and 26.5 ± 20.7 in the cytosorb group [40]. Rieder’ study suggested mean ICU length of stay was 20.5 ± 7 days in the control and 36 ± 75.5 in the cytosorb group [41]. In addition, the included studies described the severity of lung injury in the control group and the cytosorb group of the baseline. Lung injury-related indexes such as the simplified acute physiology score (SAPS-Score) decreased significantly at 72 h in the cytosorb group, but not in the control group [40]. However, the Sequential Organ Failure Assessment (SOFA) scores of lung function were 9 in both groups at baseline and decreased after organ support, but there was no significant difference between the two groups [35]. In addition, only one study focused on the difference of oxygenation index between the two groups after organ support. The oxygenation index values (12,24,48,72 h) of the two groups were significantly increased at 72 h, and it seemed to occur more rapidly in the cytosorb group, with an increasing trend at 12 h [40]. We tried to use the existing data to make forest plots to compare ECMO duration, mortality, and ICU days. However, due to the small amount of data, it is impossible to draw a convincing conclusion.

### A comparison of biological outcomes between two groups

From biological perspectives, we focused on changes in inflammatory markers and hemodynamic parameters in two groups. Inflammatory marker changes were described in six of the eight articles we included, four articles described a decrease in inflammatory factor marker levels in the cytosorb group [33, 39, 40, 42]. Two articles did not conclude a statistically significant decrease in inflammatory factor marker levels, although a decrease in inflammatory factor marker values was observed in the cytosorb group [35, 43]. Akil and colleagues ' latest study found that the level of IL-6 in the cytosorb group was significantly higher than the control group before organ support (1067.9 vs 134.8 pg/mL, p = 0.002). Compared with pre-organ support, the level of IL-6 decreased significantly after 24, 48 and 72 h of the cytosorb group, while the level of IL-6 in the control group even increased (134.8 vs 595.5 pg / ml) [40]. However, Supady and his colleagues found that the median IL-6 level in the cytosorb group decreased from 357.0 pg/mL to 98.6 pg/mL after 72 h, and the control group decreased from 289.0 pg/mL to 112.0 pg/mL. Adjusted data showed that the IL-6 levels in the cytosorb group was higher than the control group after 72 h of organ support [35]. The second study mainly found that inflammatory biomarkers such as CRP, PCT and lactic acid decreased in the blood of ARDS patients in the cytosorb group. They are markers related to sepsis diagnosis and death risk, which also illustrates the benefits of the combination of VV ECMO and hemoadsorption strategy [39]. Six of the eight articles we included described changes in hemodynamic-related drug requirements, and all of them showed that hemoadsorption reduced the dosage of related drugs and facilitated hemodynamic stability [32, 33, 35, 39–41]. The third study found that hemoadsorption reduced a large number of inflammatory factors leading to hemodynamic stability, which led to a decrease in lactic acid, vasopressor demand and an improvement in fluid balance [41]. The last study found that ECMO does not aggravate the release of cytokines in COVID-19 patients. However, the extent to which the combined use of ECMO and hemoadsorption reduces inflammation needs further study [42].

## Discussion

At present, the main strategy for patients with severe ARDS is protective lung ventilation including prone position ventilation, ECMO support and neuromuscular blockade [5, 44–46]. Hemoadsorption has been used in the treatment of patients with ARDS and has been suggested to be potentially beneficial to patients [47]. However, this conclusion is currently controversial in different studies. Our systematic review showed that hemoadsorption combined with VV ECMO may contribute to the stability of hemodynamics and reduce the demand for fluid resuscitation and vascular compression drug. Due to the high heterogeneity and low quality of the study, we could not conclude that hemoadsorption reduced the inflammatory response and mortality of ARDS patients supported by VV ECMO. It is very important that further studies are needed to clarify the role of hemoadsorption in ARDS patients supported by VV ECMO.

The eight studies we included contained only 189 patients and were all conducted in the last three years, reflecting a recent new clinical direction in the treatment of ARDS. The role of hemoadsorption in ARDS is currently unknown. Therefore, it is not appropriate to conduct large RCT studies. And only 34 patients were conducted in the only small RCT study. The adsorption device used in these studies was Cytosorb, which was listed in Europe in 2011 and approved by FDA in the United States for the treatment of COVID-19. Therefore, it is not difficult to understand that these studies were conducted in Europe and the United States. A recent systematic review suggests that although there is no hard evidence to support the use of hemoperfusion in patients with COVID-19, most studies describe a decrease in IL-6 levels after hemoperfusion [48]. A multicenter, retrospective registry from the United States showed that patients with COVID-19 (CTC) requiring ECMO in combination with CytoSorb were associated with high survival, indicating potential therapeutic benefits [43]. In addition, the ELSO registry report showed that ARDS patients treated with ECMO combined with CytoSorb had a higher 90-day ICU survival than those treated with ECMO alone [49]. However, it is regrettable that the results of the only prospective randomized controlled study show that the combination strategy has a worse therapeutic effect [35]. There are no high-quality, large randomized trials demonstrating the benefits of this combination organ support strategy. Therefore, the small, negative, randomized trial by Supady and his colleagues on whether hemoadsorption plays an important role in reducing mortality in ARDS patients was not an unexpected finding [50]. There seems to be a difference in the severity of the two groups of patients in the retrospective study. The SAPS II score of patients in cytosorb group was higher than that in the control group in two studies. The RESP and PRESERVE scores of cytosorb group patients in another study were also worse. Differences in condition among the two groups may have an impact on outcomes, especially since patients using hemoadsorption typically face a more severe inflammatory response and unstable hemodynamics. Most of the studies included in this review were COVID-19 patients, who have coagulation dysfunction and the potential activation of the coagulation system by the CytoSorb device may be a particularly relevant factor for death [51, 52]. Supady's team finds hemoadsorption and early initiation of ECMO appear to have a negative impact on patient survival [35]. However, Recent studies suggest that early use of hemoadsorption may have a positive impact on patient survival. CytoSorb has been reported to be most effective when organ support is initiated within 24 h of diagnosis of sepsis [53]. Our review could not draw any conclusions and inferences about the time of initiation, the duration of blood adsorption, and whether measuring cytokine storm is helpful. However, we find that these factors are important, which could provide direction and new insights for future investigations of hemoadsorption in ARDS patients with COVID-19 or sepsis.

Most studies in this review found that hemoadsorption may have benefits in reducing the level of inflammatory markers in ARDS patients supported by VV ECMO. It is puzzling that the statistically adjusted data from the only RCT study showed increased concentrations of IL-6 after hemoadsorption [35]. On the one hand, it is possible that the concentration of cytokines is not high enough. The level of inflammatory factors is a key factor, and high levels of inflammatory factors may be more suitable for implementing a combination organ support strategy [40]. On the other hand, there is a study that suggests the tissue cytokine concentration is much higher than the circulating. Even if the circulating cytokines are adsorbed, the intra-tissue cytokines can be rapidly replenished to the blood circulation [54]. This may explain why hemoadsorption strategies do not significantly reduce IL-6 levels in ARDS patients in some studies. Moreover, the patients' hemodynamics was improved. VV ECMO has an impact on hemodynamics and could improve respiratory acidosis and right ventricular (RV) afterload. However, we found that hemoadsorpiton reduced the use of vascular compression drugs, which is more beneficial to a rapid and sustained hemodynamic stabilization. Previous studies have shown many benefits of hemoadsorption in ECMO-supported patients with respiratory and cardiac circulatory failure, including improving hemodynamics, reducing catecholamine requirements, decreasing capillary leakage, and achieving stable metabolic parameters [20]. High levels of cytokine concentration are not always associated with high mortality. Not all diseases are suitable for the use of hemoadsorption. Therefore, we should be more cautious to use hemoadsorption widely for intensive care. Although some studies have shown that hemoadsorption could improve the patient 's condition in cardiopulmonary bypass (CPB), a systematic evaluation showed that the use of cytosorb has not reduced mortality, and it has not been confirmed that its wide application in critically ill patients is reasonable [55]. Therefore, future larger multicenter randomized prospective studies are needed to clarify which condition is suitable for the use of hemoadsorption.

The current reported case series studies on hemoadsorption have been positive, but the latest small randomized controlled trials have not had as positive results as expected [29–33, 35, 56]. The reasons for this need further study and discussion. A recent study shows that the device of hemoadsorption is nonspecific, which would affect the concentration of protective factors, but these are yet to be determined [35]. Our review showed septic shock could lead to systemic inflammatory response and ARDS. The included studies suggest that hemoadsorption may be an effective adjuvant therapy to reduce the level of circulating cytokines and regulate hemodynamics in patients with sepsis [40, 53, 57–59].

It is worth mentioning that after our screening process, several important systematic reviews and meta-analyses were published. These studies have also proved that VV ECMO combined with hemoadsorption can reduce inflammation and mortality, but most of them focus on COVID-19 patients and do not include ARDS patients induced by other causes [60, 61]. Akil and his colleagues focused on patients who was supported with VV ECMO and hemoadsorption, and observed biomarkers and clinical parameters before and after hemoadsorption, which was different from two groups in our review and limited the sample size and interpretation of the results [62].

This review summarizes clinical and biological data on VV ECMO and VV ECMO combined with hemoadsorption to help investigators focus on this clinically controversial issue and clarify the mechanisms involved. Nevertheless, we realize there are some limitations. Firstly, VV ECMO combined with hemoadsorption is currently less used in clinical ARDS disease. Most of the patients in this article were sepsis or COVID-19, which limited our interpretation and generalization of ARDS induced by other causes. Secondly, the included studies compared the effects of the two methods on patient outcomes from different perspectives, which resulted in a high heterogeneity of the results of the study. Moreover, most were retrospective studies and only one was a randomized controlled study, which may lead to high heterogeneity and selection bias of the review. Thirdly, the variability of treatment and care systems for patients with ARDS may lead to significant heterogeneity and uncertainty in the descriptions due to the lack of methods to adjust for confounding factors. Finally, due to the small number of articles and patient samples included, it is impossible to further analyze the effects of factors such as the initial time of hemoadsorption and the concentration of inflammatory factors on the therapeutic effect. In summary, a systemic review and larger clinical trials are needed.

## Conclusion

In summary, we could only draw very cautious conclusions that VV ECMO combined with hemoadsorption may improve hemodynamic stability in ARDS patients with COVID-19 or sepsis. However, the data don’t allow us to draw conclusions that hemoadsorption can reduce inflammation and mortality. Current studies and case numbers are still small and future larger multicenter randomized prospective studies are needed to clarify the role of hemoadsorption in severe ARDS patients requiring VV ECMO. The combination of ECMO and hemoadsorption may be a new strategy to reduce cytokine storm, promote lung rest, and prolong the time to the next targeted treatment for ARDS patients.

### Supplementary Information

**Additional file 1.** PRIMSA Abstract Checklist.

**Additional file 2.** PRISMA 2020 Checklist.

**Additional file 3. Table S1.** Joanna Briggs Institute Checklist.

## Data Availability

The data of this study are available from the corresponding author on reasonable request.
