# Supplementary material for: Hemoadsorption in acute respiratory distress syndrome patients requiring venovenous extracorporeal membrane oxygenation: a systematic review
Source: Respir Res. 2024 Jan 12;25:27. doi: 10.1186/s12931-024-02675-8 (PMC10785465; doi:10.1186/s12931-024-02675-8)
Supplement: Supplementary file 3 — Additional file 3. Table S1. Joanna Briggs Institute Checklist. [file 12931_2024_2675_MOESM3_ESM.docx]

| Author | Domain | | | | | | | | | | | Total  Score |
| --- | --- | --- | --- | --- | --- | --- | --- | --- | --- | --- | --- | --- |
|  | 1 | 2 | 3 | 4 | 5 | 6 | 7 | 8 | 9 | 10 | 11 |  |
| Ali Akil-2022 | × | √ | √ | √ | √ | × | √ | √ | √ | NA | √ | 8 |
| Ali Akil 2020 | √ | √ | √ | √ | √ | × | √ | √ | √ | NA | √ | 9 |
| M. Rieder2021 | √ | √ | √ | √ | √ | × | √ | √ | √ | NA | √ | 9 |
| Alexander Supady 2021 | √ | √ | √ | √ | √ | √ | √ | √ | √ | NA | √ | 10 |
| Guillaume Lebreton 2021 | × | × | √ | √ | × | √ | √ | √ | √ | NA | √ | 7 |
| Klaus Kogelmann 2020 | × | × | √ | √ | √ | √ | √ | √ | √ | NA | √ | 8 |
| Tae Song 2021 | × | × | √ | √ | √ | √ | √ | √ | √ | NA | √ | 8 |
| Travis C. Geraci MD 2021 | × | × | √ | √ | √ | √ | √ | √ | √ | NA | √ | 8 |

*Domains:*

1: Were the two groups similar and recruited from the same population?

2: Were the exposures measured similarly to assign people to both exposed and unexposed groups?

3: Was the exposure measured in a valid and reliable way?

4: Were confounding factors identified?

5: Were strategies to deal with confounding factors stated?

6: Were the groups/participants free of the outcome at the start of the study (or at the moment of

exposure)?

7: Were the outcomes measured in a valid and reliable way?

8: Was the follow up time reported and sufficient to be long enough for outcomes to occur?

9: Was follow up complete, and if not, were the reasons to loss to follow up described and explored?

10: Were strategies to address incomplete follow up utilized?

11: Was appropriate statistical analysis used?
